# Amino Acid and Biogenic Amine Profile Deviations in an Oral Glucose Tolerance Test: A Comparison between Healthy and Hyperlipidaemia Individuals Based on Targeted Metabolomics

**DOI:** 10.3390/nu8060379

**Published:** 2016-06-21

**Authors:** Qi Li, Wenbo Gu, Xuan Ma, Yuxin Liu, Lidan Jiang, Rennan Feng, Liyan Liu

**Affiliations:** Department of Nutrition and Food Hygiene, National Key Discipline, Public Health College, Harbin Medical University, Harbin 150086, China; liqi@163.com (Q.L.); guwenbo@163.com (W.G.); maxuan1@163.com (X.M.); yuxinliu@163.com (Y.L.); lidanjiang@163.com (L.J.)

**Keywords:** oral glucose tolerance test, profiles, amino acids, insulin resistance, HLP

## Abstract

Hyperlipidemia (HLP) is characterized by a disturbance in lipid metabolism and is a primary risk factor for the development of insulin resistance (IR) and a well-established risk factor for cardiovascular disease and atherosclerosis. The aim of this work was to investigate the changes in postprandial amino acid and biogenic amine profiles provoked by an oral glucose tolerance test (OGTT) in HLP patients using targeted metabolomics. We used ultra-high-performance liquid chromatography-triple quadrupole mass spectrometry to analyze the serum amino acid and biogenic amine profiles of 35 control and 35 HLP subjects during an OGTT. The amino acid and biogenic amine profiles from 30 HLP subjects were detected as independent samples to validate the changes in the metabolites. There were differences in the amino acid and biogenic amine profiles between the HLP individuals and the healthy controls at baseline and after the OGTT. The per cent changes of 13 metabolites from fasting to the 2 h samples during the OGTT in the HLP patients were significantly different from those of the healthy controls. The lipid parameters were associated with the changes in valine, isoleucine, creatine, creatinine, dimethylglycine, asparagine, serine, and tyrosine (all *p* < 0.05) during the OGTT in the HLP group. The postprandial changes in isoleucine and γ-aminobutyric acid (GABA) during the OGTT were positively associated with the homeostasis model assessment of insulin resistance (HOMA-IR; all *p* < 0.05) in the HLP group. Elevated oxidative stress and disordered energy metabolism during OGTTs are important characteristics of metabolic perturbations in HLP. Our findings offer new insights into the complex physiological regulation of metabolism during the OGTT in HLP.

## 1. Introduction 

Hyperlipidemia (HLP) is defined as a disorder of lipid metabolism that leads to abnormal increases in triglycerides (TG), total cholesterol (TC), low-density lipoprotein cholesterol (LDL-c), very low-density lipoprotein cholesterol (VLDL-c), and decreases in high-density lipoprotein cholesterol (HDL-c) [1]. HLP is a primary risk factor for the development of cardiovascular disease and atherosclerosis [2], and it has become a public health concern throughout the world. Moreover, HLP is strikingly common in patients with type 2 diabetes [3], and disturbances in lipid metabolism appear to be an early event in the development of diabetes that potentially precede disease onset by several years [4]. Therefore, a better tool is needed to monitor the disease. With the development of many high-throughput measurement technologies, metabolomics has been applied to investigate the metabolic changes of HLP. 

Metabolomics is the quantitative measurement of the dynamic multi-parametric metabolic responses of living systems to pathophysiological stimuli or genetic modifications [5]. Recently, an increasing number of metabolomics studies have been conducted to characterize hyperlipidemia models and to assess phytochemical treatment [6,7,8,9,10]. Miao et al. has reported perturbations of fatty acid, amino acid, nucleoside, and bile acid metabolisms in rats with diet-induced hyperlipidemia [6]. In those studies, beta-hydroxybutyrate, tyrosine and creatinine were found to be important biomarkers in the diagnosis of HLP [7]. These findings suggested that metabolic alterations occurred in HLP and that amino acids are closely related to HLP based on metabolomics. However, these studies were performed with fasting serum or urine in animal models, and postprandial changes in metabolism in a human study could contribute to the understanding of the physiological function of the body. Therefore, it is necessary to investigate the potential effects of postprandial metabolic changes in the amino acid profiles of HLP subjects. 

The oral glucose tolerance test (OGTT) consists of a standardized meal of pure carbohydrates and has been used to investigate postprandial variations. Several human studies have used the OGTT to investigate metabolic responses to this carbohydrate challenge based on metabolomics [11,12]. Thus, to investigate metabolic changes in the physiological responses of HLP subjects during an OGTT, we analyzed the serum amino acid and biogenic amine profiles using an ultra-high-performance liquid chromatography-triple quadrupole mass spectrometry (UPLC-TQ-MS) targeted metabolomics approach. We aimed to determine the metabolic changes influenced by this metabolic carbohydrate challenge and to explore the associations of amino acid and biogenic amine profiles with insulin resistance (IR) and thereby open new perspectives and reveal holistic regulations involved in the mechanisms of the study of the physiological reactions of HLP subjects to glucose ingestion. 

## 2. Subjects and Methods 

### 2.1. Subjects

This study was approved by the Ethics Committee of Harbin Medical University (the ethic code: 2012056) and was conducted in accordance with the Declaration of Helsinki. Written informed consent was obtained from each participant. 

All subjects were recruited from the Hexing district in Harbin city of Heilongjiang in northern China via posters in the district. A clinical examination was conducted, and anthropometric, health and lifestyle information were collected. Hyperlipidemia was diagnosed according to the levels of TG and TC. The cutoff values for TG and TC were 1.7 mmol/L and 5.7 mmol/L, respectively. The exclusion criteria were diabetes, current treatment with anti-inflammatory or other medications (especially hyperlipidemia medication), serious illness, and clinical or biochemical evidence of acute or chronic infection. Finally, thirty-five subjects with hyperlipidemia were selected. Additionally, 35 healthy adults composed the healthy control group according to the criteria of not exhibiting any significant differences in demographic characteristics, glucose or blood pressure between groups. 

Data on the physical activity levels (PALs), dietary intakes, smoking and drinking statuses were collected using face-to-face questionnaires that were answered by the participants as described in our previous study [13]. The participants who reported current alcohol consumption or smoking (at least once per month) were defined as drinkers or smokers. 

Moreover, we tested the findings for validation in the independent HLP samples (*n* = 30, age = 45.64 ± 4.81 years). These subjects were recruited from the Hexing district in Harbin city of Heilongjiang in northern China (Harbin, China).

### 2.2. Oral Glucose Tolerance Test (OGTT)

After fasting for 12 h, all subjects were challenged with the equivalent of 75 g of anhydrous glucose dissolved in 250 mL of water (OGTT). Subjects remained at rest throughout the test. Because participants in this study did not agree to be collected extra blood samples from two time points, blood samples at 0 and 120 min during an OGTT were collected. All collected blood samples were centrifuged at low speed, and serum was stored at −80 °C. 

### 2.3. Biochemical Measurements 

Serum glucose, total cholesterol (TC), LDL cholesterol (LDL-c), HDL cholesterol (HDL-c) and triglycerides (TG) were determined with kits purchased from Biosino Biotechnology (Beijing, China), standard enzymatic colorimetric techniques and with an auto-analyzer (MOL-300, Beijing, China). Serum insulin was measured with an auto-analyzer using commercial kits (Centaur, Bayer Corporation, Bayer Leverkusen, Germany). HOMA-IR was calculated according to the following equation: fasting insulin (mU/L) × fasting glucose (mmol/L)/22.5.

### 2.4. Serum Preparation for the Amino Acid Profiles

The serum amino acids and biogenic amines were prepared as previously described [14]. Briefly, each 50-µL serum sample was used for metabolite extraction before UPLC-TQ-MS analysis. The metabolite extraction procedure was performed after adding 250 µL of acetonitrile/methanol/formic acid (74.9:24.9:0.2 *v/v/v*) containing two additional stable isotope-labelled internal standards for valine-d8 and phenylalanine-d8 in the serum. After vortexing for 1 min, the mixture was maintained at room temperature for 10 min and centrifuged at 14,000 *g* for 10 min at 4 °C. The supernatant was transferred to the vial tube. The solution was filtered through a syringe filter (0.22 μm) and placed into the sampling vial for subsequent UPLC-TQ-MS analysis.

### 2.5. UPLC-TQ-MS Analysis 

UPLC-TQ-MS analysis was performed using a Waters ACQUITY UPLC system (Waters Corporation, Milford, MA, USA) coupled to a Waters Xevo TQD Mass Spectrometer (Waters Corporation, Manchester, UK). A 2 μL aliquot of the sample solution was injected into an ACQUITY UPLC™ HILIC column (100 mm × 2.1 mm i.d., 1.7 μm; Waters Corporation, Milford, MA, USA). The flow rate of the mobile phase was 300 μL/min. Analytes were eluted from the column with a gradient elution (A (10 mM ammonium formate and 0.1% formic acid, *v/v*) and B (acetonitrile with 0.1% formic acid, *v/v*)). The optimized conditions for the UPLC separation and ESI-TQ-MS detection are shown in Table S1. 

MS analyses were carried out using electrospray ionization (ESI) and multiple reaction monitoring (MRM) scans in the positive ion mode. Cone voltage and collision energies were optimized for each transition was 30 ms, the ion spray voltage was 3.2 kV, and the source temperature was 150 °C. Internal standard peak areas were monitored for quality control and individual samples with peak areas differing from the group mean by more than two standard deviations were reanalyzed. MarkerLynx Application Manager software (Version 4.1; Waters Corporation, Milford, MA, USA) was used for automated peak integration and metabolite peaks were manually reviewed for quality of integration and compared against a known standard to confirm identity.

### 2.6. Statistical Analysis

All data were presented as means ± SD. Multivariate statistical analysis was performed using SIMCA-P 11.5 software (Umetrics, Umeå, Sweden). Principal component analysis (PCA) was used first in all samples to observe the general separation. Partial least-squares-discriminant analysis (PLS-DA) was used to discriminate metabolite patterns between the OGTT time points. 

Statistical analysis was performed using SPSS 13.0 (SPSS, Inc, Chicago, IL, USA). Comparisons between HLP and healthy control were assessed with student’s *t* test for continuous variables. Within subject contrasts were used to compare values during the OGTT with zero-time values with the paired *t* test. Spearman correlation analysis was performed for the whole group using the percent change in metabolites from fasting to the 2-h sample and clinical parameters. Two-sided tests of significance were used, and a *p* value of less than 0.05 was considered to be statistically significant. 

## 3. Results 

### 3.1. Demographic and Biochemical Characteristics 

The demographic details and basic biochemical characteristics of the healthy control and HLP subjects are provided in Table 1. There were no significant differences between the two groups in terms of age, sex, body mass index, or blood pressure. The fasting blood glucose and 2-h post-challenge blood glucose levels did not differ significantly between the two groups. As expected, the TG, TC and LDL-c levels in the HLP subjects were significantly higher than those in the healthy control group (*p* < 0.001), whereas the HDL-c level of the HLP subjects was lower than that of the healthy control group (*p* < 0.001). When compared with the healthy controls, significant differences in insulin and the 2-h insulin and HOMA-IR values were observed in the HLP group (Table 1, *p* < 0.001). Moreover, there were no significant differences between the two groups regarding daily diet, physical activity level, or smoking or alcohol consumption (Table 1, *p* > 0.05). 

### 3.2. The Amino Acid and Biogenic Amine Profiles at Baseline

We examined the serum amino acid and biogenic amine profiles using UPLC-TQ-MS in the HLP and the healthy control groups. Thirty-two metabolites were detected in the fasting sera of all of the subjects (Table 2). The levels of 21 metabolites changed significantly in the HLP group compared with the healthy control group. Compared with the healthy controls, eighteen amino acids and biogenic amines were significantly increased (alanine, arginine, glutamic acid, isoleucine, leucine, lysine, phenylalanine, proline, serine, tryptophan, threonine, tyrosine, valine, creatinine, cotinine, niacinamide, thyroxine, and l-α-glycerophosphorylcholine, *p* < 0.05), and three metabolites, i.e., glutamine, γ-aminobutyric acid and taurine, were decreased (*p* < 0.05). 

**Figure 1:**
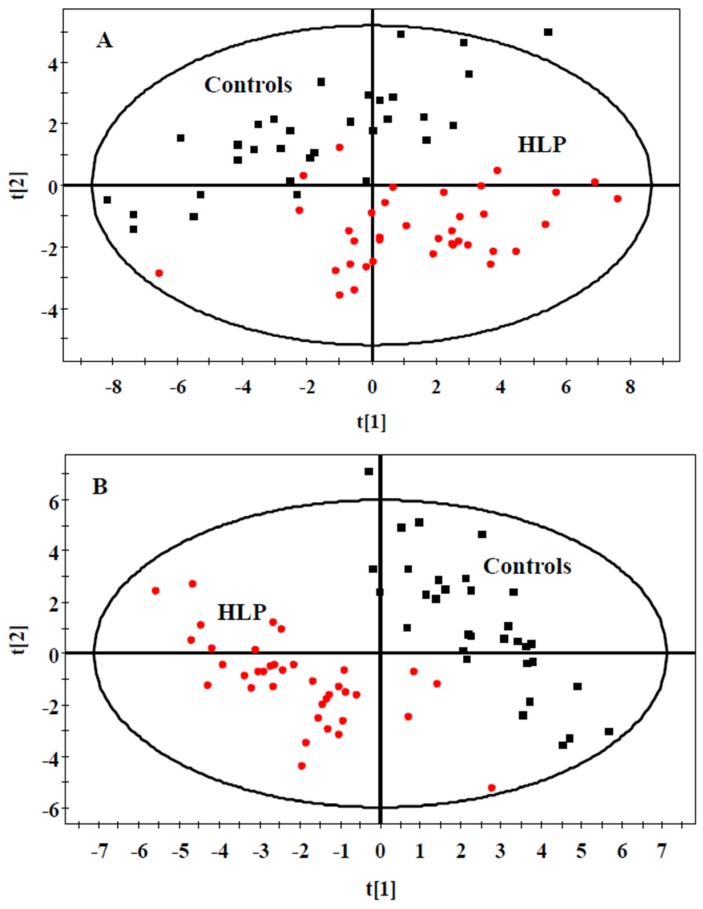
PCA (**A**) and PLS-DA (**B**) scores plot generated from serum samples data sets of the HLP and the healthy control groups. Squares (black), healthy control; Circles (red), HLP. Pprincipal component analysis (PCA), Partial least squares discriminate analysis (PLS-DA).

We also analyzed the data using multivariate statistical analysis. A plot of the PCA scores from all samples revealed a separation between the HLP and the healthy control groups (Figure 1A). The results of PLS-DA (Figure 1B) revealed a distinct separation between the two groups. These results suggested that the HLP and the healthy control groups exhibited different metabolic profiles.

### 3.3. The Amino Acid and Biogenic Amine Profile Changes during the OGTT 

**Figure 2:**
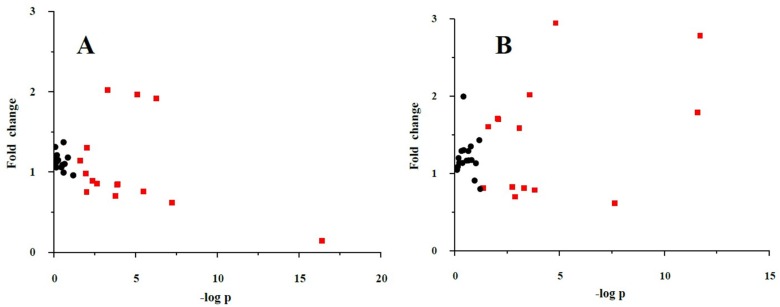
Fold change and significance of metabolite change during an oral glucose challenge in the control (**A**) and the HLP (**B**) groups. Dots represent the 32 metabolites detected in serum. Change is with respect to the fasting metabolite levels. Significant (*p* < 0.05) changes are colored red. Fold changes and percent changes for the metabolites (X) detected by UPLC-TQ-MS were calculated as follows: X_Fold change_ = (X_Concentration at 120 min_/X_Concentration at baseline_).

**Figure 3:**
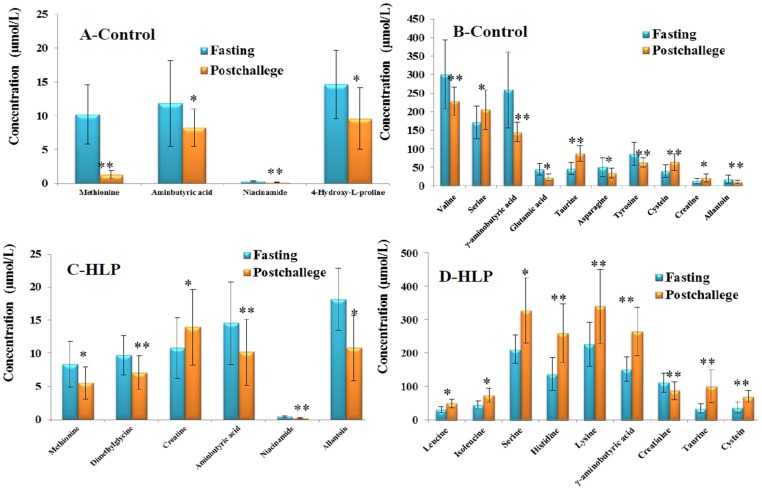
(**A**,**B**) Fourteen significant metabolites concentrations (Mean ± SD) in control group between the fasting (Blue) and post-challenge (Orange); (**C**,**D**) Fifteen significant metabolites concentrations (Mean ± SD) in control group between the fasting (Blue) and post-challenge (Orange). * *p* < 0.05, ** *p* < 0.01, compared with the control group using the paired *t* test.

**Figure 4:**
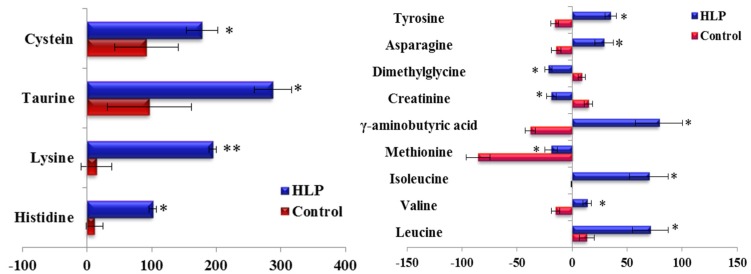
Percent change of metabolites from fasting to 2-h samples during an OGTT in the control (Blue) and HLP groups (Red). Percent changes for the metabolites (X) detected by UPLC-TQ-MS were calculated as follows: X_Percent change_ = (X_Concentration at different time (120)_ − X_Concentration at baseline_)/(X_Concentration at baseline_). * *p* < 0.05, ** *p* < 0.01, compared with the control group using student’s *t* test.

The fold-changes and significances of the metabolite changes during the OGTT between the healthy control and HLP groups are illustrated in Figure 2, Figure 3 and Figure 4. Compared with the baseline levels, the levels of 15 metabolites in the HLP group and 14 metabolites in the healthy control group were significantly affected by the OGTT (Figure 2), but different trends in the metabolites changes were observed between the two groups. In the healthy controls, the levels of ten metabolites (methionine, aminobutyric acid, niacinamide, 4-hydroxy-l-proline, valine, γ-aminobutyric acid (GABA), glutamic acid, asparagine, tyrosine and allantoin) decreased, and the concentrations of four amino acids (serine, taurine, cysteine and creatine) increased significantly after the OGTT (Figure 3A,B). However, there were significant increases in nine metabolites (leucine, isoleucine, serine, histidine, lysine, γ-aminobutyric acid, taurine, cysteine and creatine) and reductions in six metabolites (methionine, dimethylglycine, aminobutyric acid, niacinamide, allantoin and creatinine) in the HLP group after the OGTT (Figure 3C,D). When compared with the healthy controls, the per cent changes in 13 metabolites in the HLP group were significantly different (Figure 4). There were inverse variations in 7 metabolites between the two groups. Specifically, the per cent change in γ-aminobutyric acid was −37.9% in the healthy control after glucose loading, while the per cent change in the HLP subjects was 79.1% (*p* < 0.001).

**Figure 5:**
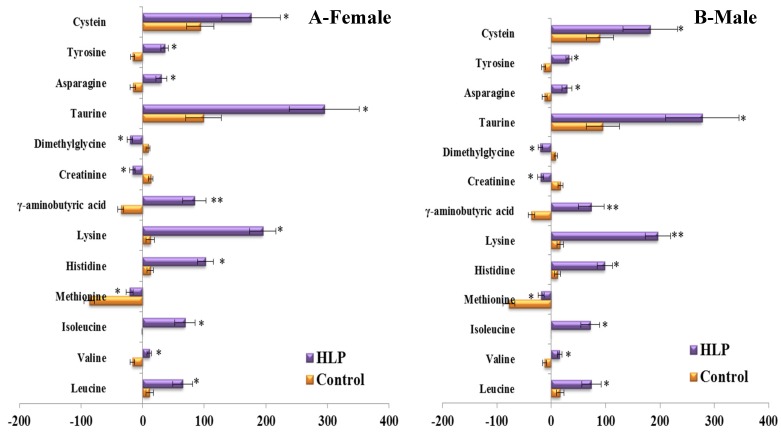
Percent changes of metabolites from fasting to 2-h samples during an OGTT between female (**A**) and male (**B**) in the control (Orange) and HLP groups (Purple). Percent changes for the metabolites (X) detected by UPLC-TQ-MS were calculated as follows: X_Percent change_ = (X_Concentration at different time (120)_ − X_Concentration at baseline_)/(X_Concentration at baseline_). * *p* < 0.05, ** *p* < 0.01, compared with the control group using student’s *t* test.

**Figure 6:**
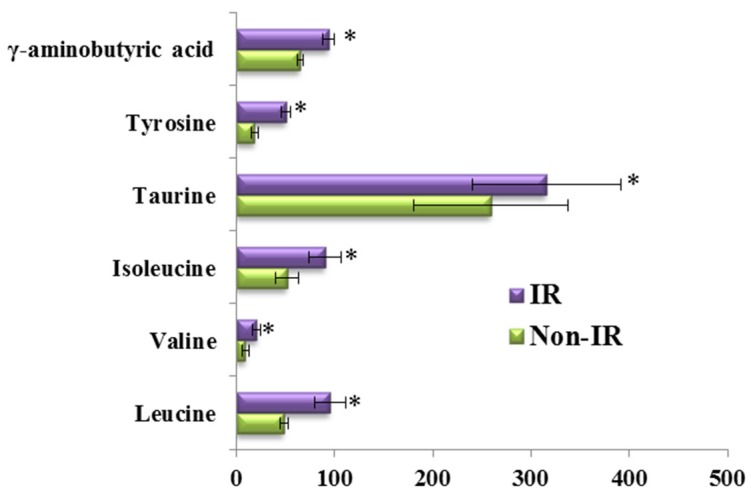
Percent changes of metabolites from fasting to 2-h samples during an OGTT between subjects with IR (Purple) and without IR (Green) in the HLP group. Percent changes for the metabolites (X) detected by UPLC-TQ-MS were calculated as follows: X_Percent change_ = (X_Concentration at different time (120)_ − X_Concentration at baseline_)/(X_Concentration at baseline_). * *p* < 0.05, compared with the control group using student’s *t* test.

To consider the effect of gender, we compared the differences in the responses of the amino acid and biogenic amine profiles to the OGTT between men and women in both groups (Figure 5). Consistent with the above results, the per cent changes in 13 metabolites during the OGTT in the HLP group were significantly different from those in the healthy control group when we analyzed the data from the female subjects in both groups (Figure 5A). The same results were also observed in the male subjects of both groups (Figure 5B). Moreover, we also analyzed the differences in the responses of the amino acid and biogenic amine profiles to the OGTT between the subjects with and without IR in the HLP group. In this work, IR was defined as a HOMA-IR higher than 2.50 [15]. The HOMA-IR values of 11 subjects are lower than 2.5, and 24 subjects exhibited insulin resistance. Our results revealed that the per cent changes in 6 metabolites (γ-aminobutyric acid, tyrosine, taurine, isoleucine, leucine, and valine) in the IR group were greater than those in the non-IR subjects (Figure 6). 

### 3.4. Correlations between the Clinical Parameters and the Per Cent Changes from the 2-h Minus Fasting Metabolite Responses to the OGTT

Clinical parameters, such as TG, TC, LDL-c, HDL-c, fasting insulin, 2-h insulin and HOMA-IR, were associated with changes in metabolite levels (Table 3). In the healthy control group, the TC was negatively associated with changes in valine and isoleucine between 0 and 120 min (*p* < 0.05), and TG was positively related to changes in valine, serine and creatine. HDL-c was negatively correlated with the changes in dimethylglycine and lysine (*p* < 0.05), while LDL-c was positively associated with the postprandial changes in serine and creatinine (*r*: 0.35 and 0.379, respectively, *p* < 0.05). Moreover, the 2-h insulin was negatively correlated with histidine (*r*: −0.355, *p* < 0.05).

Regarding the HLP group, TC was positively associated with changes in valine, serine, isoleucine and asparagine between 0 and 120 min during the OGTT (*p* < 0.05). Increasing TG levels were positively associated with changes of several metabolites including valine, serine, isoleucine, creatine asparagine and tyrosine (all *p* < 0.05), while HDL-c was negatively associated with six metabolites in response to the OGTT, i.e., valine, isoleucine, creatinine, dimethylglycine, asparagine and tyrosine (all *p* < 0.05). LDL-c was positively related to the changes in five metabolites (valine, serine, creatinine, dimethylglycine, and tyrosine). Regarding insulin, isoleucine was positively correlated with the fasting insulin (*r*: 0.371, *p* = 0.037), and histidine was negatively associated with the 2-h insulin (*r*: −0.355, *p* < 0.05). Additionally, the HOMA-IR was significantly correlated with the changes in isoleucine and γ-aminobutyric acid (GABA) during the OGTT (all *p* < 0.05; Table 3). 

### 3.5. Validation of the Significant Metabolites

**Figure 7:**
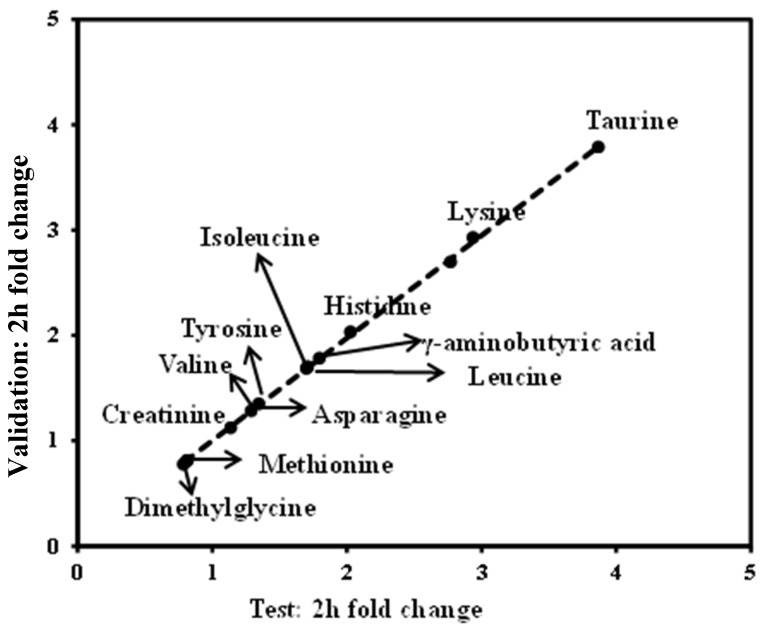
Validation of metabolite changes at the 2-h time point. In total, 13 metabolites that changed significantly in the test study (Figure 4) replicated (*p* < 0.05) in the validation study. Dots correspond to the median fold change of metabolites at the 2-h time point. Fold changes for the metabolites (X) detected by UPLC-TQ-MS were calculated as follows: X_Fold change_ = (X_Concentration at 120 min_/X_Concentration at baseline_). The arrows indicate the metabolisms in the test and validation set.

To confirm these findings, we determined the fasting and 2-h OGTT sample profiles from an independent study. This group exhibited similar TC and TG values relative to the HLP group that was defined as the test set. Of the 13 metabolites that exhibited significant changes (*p* < 0.05), in the test set, at the 2-h time point during the OGTT (Figure 7), similar trends were also observed in the validation group. These metabolite changes were significantly replicated (*p* < 0.05) and are demonstrated in Figure 7. Thus, we identified 13 serum metabolites that exhibited highly reproducible and robust responses to glucose ingestion in the HLP subjects. 

## 4. Discussion

In this study, we characterized 32 metabolites during a standard 2-h OGTT in the HLP and the healthy control groups using a targeted metabolomics approach. To our knowledge, this is the first study to apply profiling technology to characterize the responses of the amino acid and biogenic amine profiles during OGTT in HLP subjects. Regarding the baseline differences, there was low taurine in the HLP group compared with the healthy control (*p* < 0.05). Moreover, the levels of sulfur amino acids (i.e., taurine and cysteine) in the postprandial state were significantly increased in the HLP group compared with the fasting state (Figure 3). Several studies have demonstrated that the pathophysiology of postprandial deregulated metabolism, especially hyperglycaemia, is characterized by hyperglycaemic spikes that induce oxidative stress [16,17]. In our previous work, we also found that a glucose overload resulted in a significant fall in the SOD and GSH-Px concentrations in the HLP subjects [18]. Taurine, which is a sulfuric amino acid, can be synthesized by the human body from cysteine. Taurine has many diverse biological functions that serve as stabilizers of cell membranes and anti-oxidants. Our data demonstrated that increased cysteine and taurine occur during OGTTs in HLP subjects. These findings are similar to those of another study that reported a relative increase in the concentration of taurine after drinking a simple sugar solution [19]. The high cysteine and taurine during the OGTT in the HLP subjects may represent a reaction that functions to resist the status of oxidative stress during the OGTT because of the anti-oxidation function of these amino acids. 

Serum branched-chain amino acids (BCAAs; i.e., isoleucine, leucine, and valine) and aromatic amino acids (phenylalanine) in the fasting state were significantly increased in the HLP group compared with the healthy controls (Table 2). BCAAs are essential amino acids in humans and play central roles in protein metabolism [20], that include improving glucose metabolism [21] and regulating leptin secretion during food intake [22]. Newgard et al. [23] recently demonstrated that BCAAs contribute to insulin resistance (IR) and that high concentrations of BCAAs can lead to insulin resistance [24]. Moreover, elevated levels of BCAAs have been reported to be strongly associated with the future risk of diabetes [25]. In this work, we observed an increase in BCAAs during the OGTTs in the HLP groups (Figure 3), and the postprandial changes in these amino acids were validated in our study (Figure 7). Therefore, it is necessary to explore the associations between postprandial changes in BCAAs and IR. 

Although the idea that BCAAs and several related amino acids are linearly related to HOMA-IR has been supported by some studies [23,26], few studies have investigated the relationship between the postprandial changes in amino acids and the HOMA-IR. Our data revealed that there were positive associations between the postprandial changes in isoleucine and HOMA-IR (Table 3). Furthermore, we also found that the BCAAs were positively correlated with lipid parameters (TG, TC, LDL-c, HDL-c). In animal experiments, it has been reported that an interaction between excess high fat and BCAAs in development of insulin resistance is mediated by impaired insulin signaling in the liver and the muscles [23]. Moreover, Newgard developed a model to explain the interplay between lipids and branched-chain amino acids in the development of insulin resistance [27]. Therefore, our results suggest that the postprandial changes in BCAAs may shed new light on the metabolic deregulation associated with IR in HLP. 

Notably, correlations between the postprandial changes in creatine, creatinine, dimethylglycine, asparagine, serine, tyrosine and lipid indices (TC, TG, HDL-c and LDL-c) were found in the HLP subjects. These results suggest that the postprandial changes in these metabolites may be used as biomarkers in HLP subjects. Changes observed in some metabolites such as creatine, creatinine, and asparagine suggest disordering of energy metabolism. Creatine plays a fundamental role in energy buffering and the overall cellular bioenergetics by means of the creatine kinase/phosphocreatine system, which is responsible for the transfer of energy from the mitochondria to the cytosol [28]. In contrast, the increases in the serum levels of fasting creatinine in the HLP group (Table 2), which is a non-enzymatic degradation product of creatine and phosphocreatine, also support the hypothesis that abnormal metabolism of creatine is associated with the development of HLP. Moreover, asparagine is a precursor of many other amino acids, such as aspartate, glutamine and glutamate, which can be used to supply energy to enterocytes. Wang et al. indicated that asparagine supplementation improves energy status [29] and attenuates the changes in serum biochemical parameters in weaned piglets after the administration of a lipopolysaccharide challenge [30]. In this work, the postprandial change in asparagine was positively related to TG and TC, which may be hint that a postprandial disorder of energy metabolism occurred. Thus, it may be of interest to investigate the mechanism of the postprandial changes in the energy metabolism in HLP patients, which may be helpful in improving HLP.

One remarkable observation in this work is that the serum fasting GABA was down-regulated in the HLP subjects compared with the controls. The postprandial GABA significantly increased in the HLP subjects after glucose loading (ΔGABA, 79.06%), whereas a postprandially low level of γ-aminobutyric acid was observed in the controls (ΔGABA, −39.95%; Figure 4). The HOMA-IR was significantly correlated with the changes in GABA during the OGTT (*p* < 0.05) (Table 3). GABA is produced from glutamic acid by glutamic acid decarboxylase. In the healthy controls, there were low levels of postprandial glutamic acid (Figure 3) compared with the baseline. Therefore, the low glutamic acid level may have contributed to the postprandial decrease in GABA in the healthy controls. Moreover, in the pancreas, GABA is produced primarily by insulin-secreting beta cells [31], and the release of GABA from these cells appears to be regulated by glucose [32]. In our previous study, we found that there were different postprandial glucose profiles during OGTTs between HLP and control groups [33]. Therefore, the different postprandial glucose profiles in the two groups might have contributed to the different postprandial changes in the GABA levels. Moreover, GABA had anti-oxidative effects [34], and acute hyperglycemia after a meal or glucose load increases oxidative stress [35]. Thus, the increased GABA in the HLP subjects might have been a stress response to ameliorate or prevent high postprandial oxidative stress in HLP individuals. 

There are a number of limitations to the present study. First, the relevance of the changes in the postprandial amino acid and biogenic amine profiles in the molecular mechanism of HLP could not be explained. Therefore, animal and cellular experiments should be performed in the future. Second, the duration of the postprandial investigation was short (only 2 h); thus, the results are relevant only to the short-term effects. Third, the number of subjects studied was relatively small, and the included subjects did not represent a random sample of the Chinese population; thus, caution is required in generalizing the results of this study to the entire Chinese population. Therefore, additional studies in this research field are needed in the future.

## 5. Conclusions

In conclusion, our study found that the amino acid and biogenic amine profiles differed between HLP individuals and healthy controls at baseline and after an OGTT. Elevated fasting and postprandial levels of the BCAAs after a single carbohydrate challenge revealed significant metabolic alterations in the amino acids. Elevated oxidative stress and postprandial disorders in energy metabolism during OGTTs are important characteristics of metabolic perturbations in HLP. The correlation between postprandial changes in BCAAs and GABA with IR in HLP may facilitate our understanding of the functions of amino acids in HLP. Our findings offer new insights into the complex physiological regulation of metabolism during OGTT in HLP. 

## Figures and Tables

**Table 1:** Demographic and clinical chemistry characteristics of human subjects.

Parameters	Control (*n* = 35)	HLP (*n* = 35)	*p* Value
Sex (female/male)	17/18	16/19	0.12
Age (years)	47.54 ± 10.08	48.21 ± 8.24	0.11
Smoker/non-smoker	16/19	15/20	0.45
Alcohol consumption (%)	32.45	33.12	0.56
Protein (g/day)	61.34 ± 12.23	60.18 ± 14.31	0.67
Fat (g/day)	57.25 ± 10.04	56.94 ± 10.02	0.48
Carbohydrate (g/day)	302.45 ± 56.17	301.34 ± 58.28	0.51
Physical activity level	1.42 ± 0.34	1.37 ± 0.42	0.41
BMI (kg/m^2^)	22.30 ± 1.72	23.16 ± 1.68	0.32
TC (mmmol/L)	4.14 ± 0.53	6.41 ± 0.61	<0.001
TG (mmmol/L)	0.94 ± 0.31	2.69 ± 0.83	<0.001
HDL-c (mmol/L)	1.34 ± 0.18	1.00 ± 0.12	<0.001
LDL-c (mmol/L)	2.51 ± 0.45	4.21 ± 0.76	<0.001
Fasting glucose (mmmol/L)	4.08 ± 0.49	4.18 ± 0.47	0.08
2 h-glucose (mmmol/L)	4.65 ± 0.94	5.01 ± 0.68	0.06
SBP (mmHg)	113.15 ± 6.31	115.68 ± 8.99	0.19
DBP (mmHg)	75.69 ± 6.23	79.15 ± 8.25	0.09
Fasting insulin (mU/L)	6.86 ± 3.07	13.91 ± 2.95	<0.001
2h-insulin (mU/L)	6.82 ± 2.14	36.72 ± 6.85	<0.001
HOMR-IR	1.19 ± 0.36	2.82 ± 0.66	<0.001

HLP: Hyperlipidemia; BMI: body mass index; SBP: Systolic blood pressure; DBP: Diastolic blood pressure; TG: Triglycerides; TC: Total cholesterol. HDL-c: High-density lipoprotein cholesterol; LDL-c: Low-density lipoprotein cholesterol; FBG: fasting plasma glucose; 2 h-PG: 2 h Postprandial plasma glucose; HOMA-IR: Homeostasis model assessment of insulin resistance.

**Table 2:** Quantitative analysis of amino acids, biogenic amines and fatty acids in the fasting serum between the healthy control and HLP groups.

Metabolites (μmol/L)	Control (*n* = 35)	HLP (*n* = 35)	*p* Value
Alanine	522.80 ± 191.22	729.05 ± 164.80	<0.001
Arginine	137.85 ± 65.43	268.08 ± 79.09	<0.001
Cystein	39.42 ± 16.53	37.11 ± 20.01	0.607
Glycine	352.65 ± 123.97	365.16 ± 81.11	0.617
Glutamic acid	44.26 ± 15.47	54.73 ± 14.13	0.001
Histidine	128.31 ± 46.08	137.03 ± 48.55	0.449
Isoleucine	35.68 ± 10.84	45.58 ± 11.76	0.001
Leucine	21.39 ± 7.50	30.94 ± 8.51	<0.001
Lysine	219.68 ± 79.02	226.50 ± 65.71	<0.001
Methionine	9.98 ± 4.30	8.34 ± 3.44	0.077
Phenylalanine	119.44 ± 34.03	168.05 ± 34.36	<0.001
Proline	208.95 ± 79.25	248.31 ± 62.49	0.024
Serine	170.18 ± 43.03	211.45 ± 42.34	<0.001
Tryptophan	70.43 ± 16.72	82.66 ± 23.08	0.016
Threonine	164.72 ± 54.32	192.25 ± 39.57	0.018
Tyrosine	85.73 ± 29.39	118.24 ± 30.16	<0.001
Valine	296.90 ± 90.61	370.06 ± 86.03	0.001
Asparagine	50.57 ± 15.10	48.45 ± 17.12	0.680
Creatine	13.31 ± 7.19	10.79 ± 4.62	0.084
Creatinine	84.39 ± 27.51	111.45 ± 28.19	<0.001
Cotinine	0.039 ± 0.01	0.46 ± 0.24	<0.001
Dimethylglycine	11.26 ± 5.06	9.71 ± 2.96	0.119
Glutamine	37.32 ± 11.52	23.93 ± 13.24	0.005
Allantoin	18.46 ± 9.74	18.16 ± 4.70	0.901
4-Hydroxy-l-proline	15.53 ± 5.07	12.40 ± 6.37	0.202
Niacinamide	0.28 ± 0.09	0.42 ± 0.12	<0.001
Thyroxine	0.021 ± 0.01	0.04 ± 0.01	<0.001
Trimethylamine-*N*-oxide	0.36 ± 0.14	0.37 ± 0.20	0.859
Aminobutyric acid	11.83 ± 6.08	14.53 ± 6.23	0.075
γ-aminobutyric acid	264.56 ± 102.19	152.28 ± 35.40	<0.001
Taurine	46.38 ± 16.46	34.50 ± 13.38	0.002
l-α-Glycerophosphorylcholine	12.98 ± 4.40	17.15 ± 4.79	0.004

**Table 3:** Associations between clinical parameters and percent change of metabolites from fasting to 2-h sample response to an OGTT.

Metabolites	Control (*n* = 35)						
	TC	TG	HDL-c	LDL-c	Fasting Insulin	2h-Insulin	HOMA-IR
Valine	−0.345 (0.041)	−0.386 (0.032)					
Isoleucine	−0.347 (0.042)						
Serine		0.364 (0.034)		0.351 (0.047)			
Histidine						−0.355 (0.039)	
Creatine		0.373 (0.035)					
Lysine			−0.357 (0.045)				
Creatinine				0.379 (0.033)			
Dimethylglycine			−0.408 (0.020)				
Metabolites	HLP (*n* = 35)						
	TC	TG	HDL-c	LDL-c	Fasting insulin	2h-insulin	HOMA-IR
Valine	0.398 (0.027)	0.475 (0.006)	−0.358 (0.038)	0.351 (0.041)			
Isoleucine	0.368 (0.038)	0.387 (0.029)	−0.349 (0.042)		0.371 (0.037)		0.353 (0.047)
Serine	0.352 (0.045)	0.429 (0.014)		0.352 (0.043)			
Histidine						−0.455 (0.009)	
γ-aminobutyric acid							0.374 (0.031)
Creatine		0.473 (0.006)					
Creatinine			−0.373 (0.03)	0.409 (0.016)			
Dimethylglycine			−0.341 (0.048)	0.475 (0.004)			
Asparagine	0.441 (0.012)	0.395 (0.025)	−0.338 (0.049)				
Tyrosine		0.42 (0.017)	−0.383 (0.025)	0.368 (0.032)			

TG: Triglycerides; TC: Total cholesterol. 2h-insulin: 2 h Postprandial plasma glucose. HOMA-IR, homeostatic model of assessment-insulin resistance. Data for metabolites-clinical parameter associations which did not achieve statistical significance are not included. Data are presented as standardized correlation-coefficients (*p*-values). Standardized correlation-coefficients were computed from standard deviations with *p*-values < 0.05.

## Supplementary Materials

The following are available online at http://www.mdpi.com/2072-6643/8/6/379/s1, Table S1: UPLC-ESI-TQ-MS conditions.
