# Supplementary material for: Amino Acid and Biogenic Amine Profile Deviations in an Oral Glucose Tolerance Test: A Comparison between Healthy and Hyperlipidaemia Individuals Based on Targeted Metabolomics
Source: Nutrients. 2016 Jun 21;8(6):379. doi: 10.3390/nu8060379 (PMC4924220; doi:10.3390/nu8060379)
Supplement: Supplementary file 1 [file nutrients-08-00379-s001.docx]

Supplementary Materials: Amino Acid and Biogenic Amine Profile Deviations in an Oral Glucose Tolerance Test: A Comparison between Healthy and Hyperlipidaemia Individuals Based on Targeted Metabolomics

Qi Li, Wenbo Gu, Xuan Ma, Yuxin Liu, Lidan Jiang, Rennan Feng and Liyan Liu

**Table S1.** UPLC-ESI-TQ-MS conditions.

|  | **UPLC (Separation Conditions)** |
| --- | --- |
| Column | ACQUITY UPLC™ HILIC column |
|  | (100 mm × 2.1 mm i.d., 1.7 μm, Waters) |
| Mobile phase A | 10 mM ammonium formate and 0.1% formic acid, *v/v* |
| Mobile phase B | Acetonitrile with 0.1% formic acid, *v/v* |
| Gradient elution | A% = 5% maintained (0–0.5 min), increased to 40% in 6 min, |
|  | 50% linearly increased (6–7 min), |
|  | 50% maintained 1 min(7–8 min), followed by re-equilibration |
|  | to the initial conditions in 6 min (8–15 min). |
| Flow rate | 0.30 mL/min |
| Injection volume | 2 μL |
|  | TQ-MS Condition |
| Polarity | ESI positive |
| Capillary voltage | 3200 V |
| Desolation gas flow | 650 L/h |
| Cone gas flow | 50 L/h |
| Source temperature | 150 °C |
| Desolvation temperature | 400 °C |

**Reference**

1. Liu, L.; Feng, R.; Guo, F.; Li, Y.; Jiao, J.; Sun, C. Targeted metabolomic analysis reveals the association between the postprandial change in palmitic acid, branched-chain amino acids and insulin resistance in young obese subjects. *Diabetes Res. Clin. Pract.* **2015**, *108*, 84–93.
